# Supplementary material for: Selection of Optimal Palmer Predictors for Increasing the Predictability of the Danube Discharge: New Findings Based on Information Theory and Partial Wavelet Coherence Analysis
Source: Entropy (Basel). 2022 Sep 27;24(10):1375. doi: 10.3390/e24101375 (PMC9601209; doi:10.3390/e24101375)
Supplement: Supplementary file 1 [file entropy-24-01375-s001.zip › entropy-1820471-supplementary.pdf]

Supplementary Materials

Selection of optimal Palmer predictors for increasing the predictability of the Danube discharge: New findings based on information theory and partial wavelet coherence analysis

Ileana Mares, Constantin Mares, Venera Dobrica, Crisan Demetrescu

Table S1. Normalized mutualinformation (NMI) and the redundancy-synergy index (RSI) with 4, 3 and 2 predictors for estimating the discharge at Orsova (Q). Only situations with RSI> 0 are presented. Cases are highlighted in which relatively high RSI is also associated with a higher NMI

PALMER Indices	Q	NMI	RSI
WIN	FALL		
(PDSI,PHDI,WPLM,ZIND)		<u>0.6471</u>	<u>0.2679</u>
(PDSI,PHDI,WPLM)		<u>0.6379</u>	<u>0.1792</u>
(PDSI,PHDI, ZIND)		0.4039	0.1946
(PDSI,WPLM,ZIND)		0.4012	0.1712
(PHDI,WPLM,ZIND)		0.4746	0.1115
(PDSI,PHDI)		0.3103	0.0965
(PDSI, WPLM)		0.3193	0.0722
(PHDI,WPLM)		0.5619	0.0153
SPR	FALL		
(PDSI,PHDI,WPLM,ZIND)		<u>0.7397</u>	<u>0.2840</u>
(PDSI,PHDI,WPLM)		0.7109	0.1380
(PDSI,PHDI, ZIND)		0.5086	0.1614
(PDSI,WPLM,ZIND)		<u>0.5931</u>	<u>0.2135</u>
(PHDI,WPLM,ZIND)		0.5514	0.1820
(PDSI, WPLM)		0.5506	0.0815
(WPLM,ZIND)		0.2544	0.0853
(PHDI,WPLM)		0.5145	0.0571

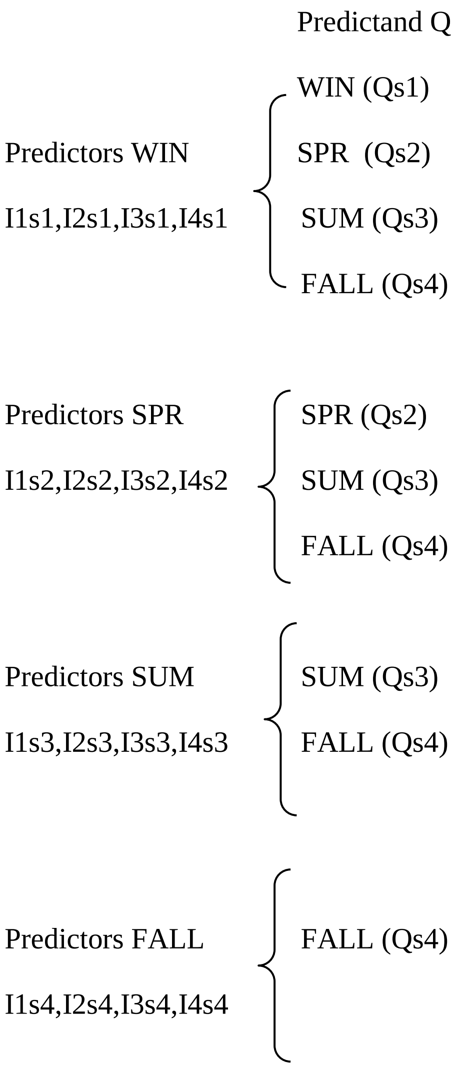

Figure S1. Schematic representation of the 10 predictor-predictand combinations.

I1, I2, I3 and I4 are associated with the Palmer Indices: PDSI, PHDI, WPLM and ZIND. s1, s2, s3 and s4 represent the Winter, Spring, Summer and Fall seasons.

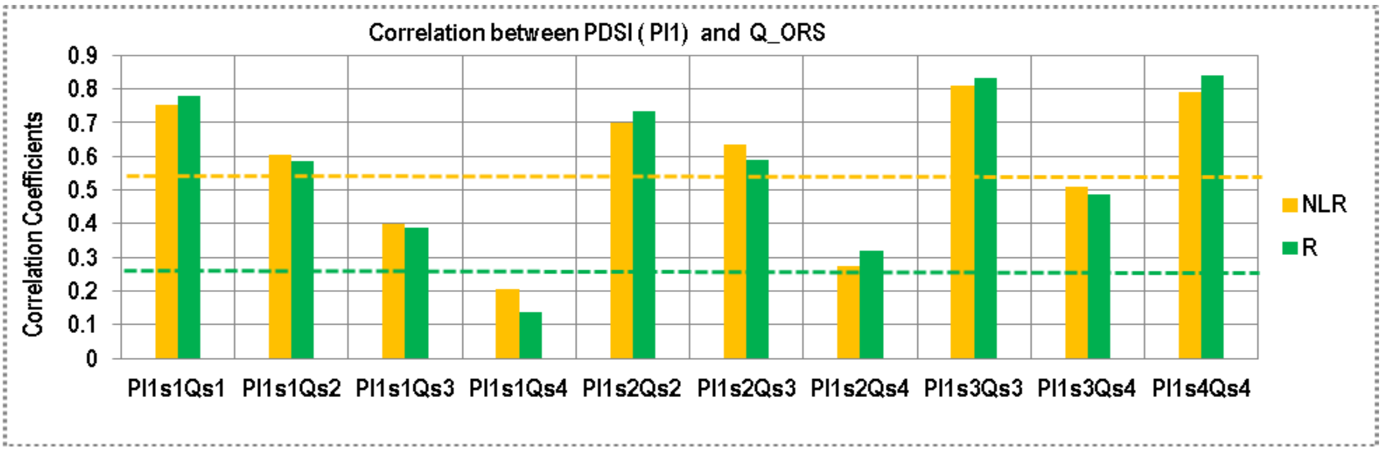

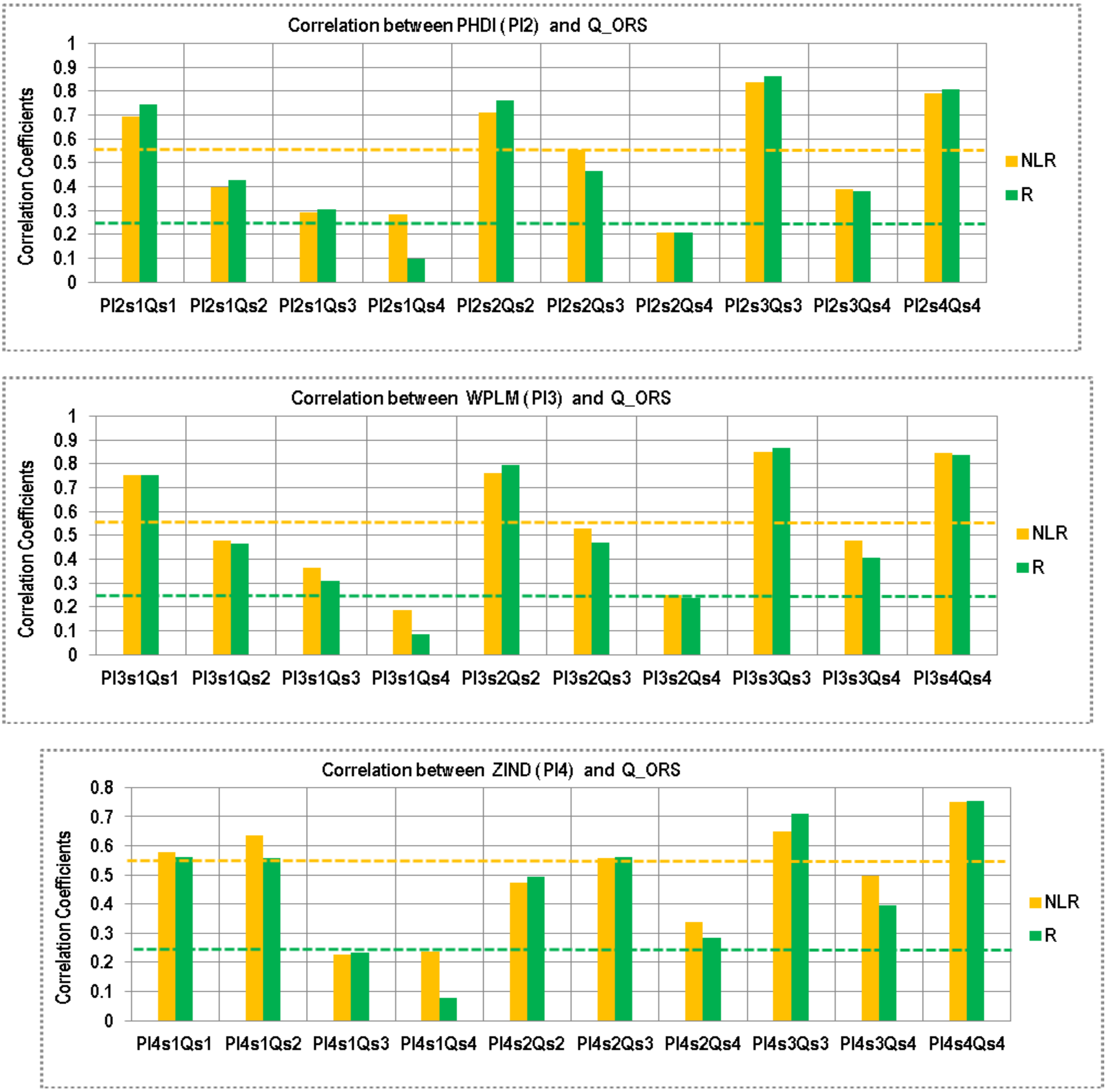

Figure S2. Linear (|R|) and nonlinear (NLR) correlation coefficients between Orsova discharge and the four predictors for ten combinations. The horizontal lines represent confidence level (CL) of 99% for the respective correlations.

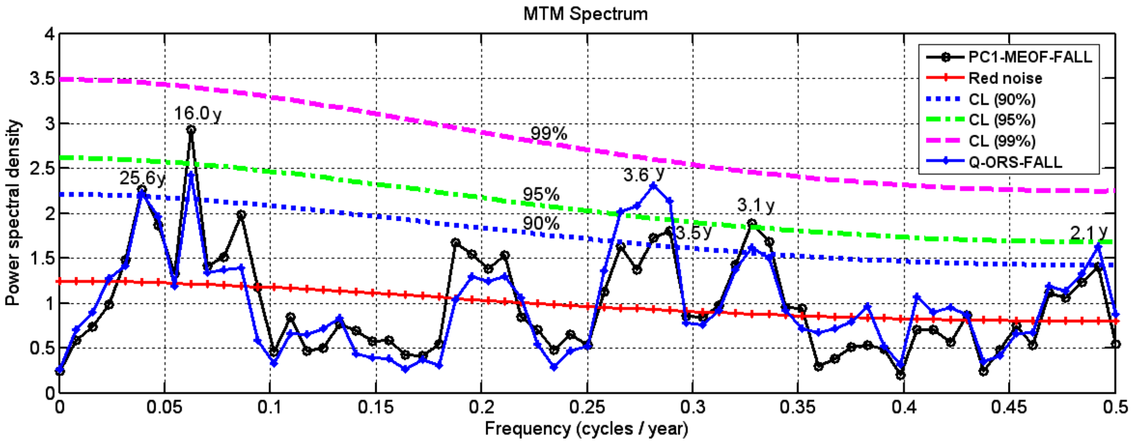

Figure S3. Power spectra for FALL, estimated by MTM, with the number of tapers K=3 for the Orsova discharge (Q-ORS) and PC1-MEOF. Both time series are standardized. (Mares et al., 2016)
